# Distribution of hounsfield unit values in the pelvic bones: a comparison between young men and women with traumatic fractures and older men and women with fragility fractures: a retrospective cohort study

**DOI:** 10.1186/s12891-022-05263-3

**Published:** 2022-03-29

**Authors:** Naoya Inagaki, Takaaki Tanaka, Jun Udaka, Shoshi Akiyama, Tatsuki Matsuoka, Mitsuru Saito

**Affiliations:** 1 Department of Orthopedic Surgery, The Jikei University School of Medicine, Tokyo, Japan; 2 Department of Orthopedic Surgery, NHO Utsunomiya National Hospital, Tochigi, Japan

**Keywords:** Pelvic fracture, Bone mineral density, Hounsfield unit, Fragility fracture

## Abstract

**Background:**

The fixation strength of bone screws depends on bone mineral density (BMD), so it is important to evaluate bone strength at fracture sites. Few studies have investigated BMD in the pelvis. The aims of this study were to measure the regional Hounsfield unit (HU) values in the cancellous bone of the acetabulum and pelvic ring and to compare these values between young and older patients.

**Methods:**

This study enrolled young patients with high-energy trauma (aged 20–44 years; young group) and older patients with low-energy trauma (aged 65–89 years; older group). Patients without pelvic computed tomography (CT) scans, those with pelvic bone implants, and those who died were excluded. The HU values on the contralateral (non-fractured) side of the pelvis were measured on CT scans. The CT data were divided into 7 areas: the pubic bone, the anterior and posterior walls and roof of the acetabulum, the ischial tuberosity, the body of the ilium, and the third lumbar vertebra. The HU values in each area were compared between the young and older groups.

**Results:**

Sixty-one young patients and 154 older patients were included in the study. The highest HU value was in the roof of the acetabulum regardless of age and sex. HU values were significantly higher in the ischial tuberosity and body of the ilium and lower in the pubic bone and anterior wall. The HU values in all pelvic areas were significantly lower in the older group than in the young group, especially in the anterior area.

**Conclusions:**

HU values in the 6 pelvic areas were not uniform and were strongly related to load distribution. The HU distribution and age-related differences could explain the characteristic causes and patterns of acetabular fractures in the older and may help in surgical treatment.

## Background

Osteoporosis is common in the elderly population and is associated with an increased risk of fragility fractures [1]. In patients over the age of 60 years, the incidence of acetabular fractures has increased 2.4-fold over the past quarter-century, and geriatric patients are the most rapidly growing subgroup of patients [2]. Epidemiologic studies suggest that approximately 4,000 acetabular fractures occur in the elderly each year in the United States [3]. Tile et al. recommended that attempts at osteosynthesis of geriatric acetabular fractures be abandoned because of the poor BMD in this age group and that nonoperative management should be considered when feasible [4]. However, several studies have shown that the results of nonoperative treatment are poor and associated with severe pain on walking or incapacity in 30% of cases [3, 5, 6]. Moreover, some researchers have found that surgery allows rapid mobilization on a walker or crutches [4, 7], and Cornell et al. reported that operative management of displaced acetabular fractures yielded better results than nonoperative methods [8]. It is important to evaluate bone strength at fracture sites when the bone reconstruction of osteoporotic pelvis is performed. Because the fixation strength of bone screws depends on BMD [9, 10]. Therefore, Identifying BMD of the pelvis may be crucial to developing better treatment options.

Dual-energy X-ray absorptiometry is the widely used method for measuring BMD [11]. However, it is difficult to measure the distribution of BMD in the pelvis by this method because of its complex shape. Furthermore, dual-energy X-ray absorptiometry often overestimates BMD due to degenerative changes and vessel calcification [12].

More recent studies have measured BMD using the Hounsfield unit (HU) values obtained on CT scans [1, 13–18]. For example, HU values at the distal radius, thoracic vertebrae, femoral head, femoral neck, and proximal humerus have been used to evaluate the BMD for diagnosis of osteoporosis [13–17], because significant correlation between BMD and HU values for each area have been found. However, although HU values have been examined in the sacrum [1, 19–21], to our knowledge, there are no reports on the HU distribution in the pelvis. The aims of this study were to measure the regional HU values in the acetabulum and pelvic ring and to compare the measurements obtained between young and older patients.

## Methods

### Patients

We enrolled young patients with pelvic fractures caused by high-energy trauma (aged 20–44 years; young group) and older patients with pelvic fractures caused by low-energy trauma (aged 65–89 years; older group) between January 1, 2015 and December 31, 2019. Low-energy trauma was defined as injury resulting from a fall from standing height and high-energy trauma as injury resulting from a road traffic accident, crush injury, or a fall from height of ≥ 5 m. Patients in whom pelvic CT was not performed, those with pelvic bone implants, those who died for religious reasons, and those who were older with high-energy trauma, with diabetes mellitus and use of corticosteroids were excluded. The BMI of older men and women was 20.2 ± 3.19 and 21.1 ± 3.75, respectively.

### Imaging

A multidetector CT system (Aquilion PRIME, Canon Medical Systems, Otawara, Japan) was used with the following parameters: tube voltage, 120 kV; automatic exposure control (AEC) with a standard deviation (SD) setting of 14 to 5-mm slice thickness soft kernel (FC13) images, helical scan mode; rotation time 0.5 s; and pitch factor 0.813. The CT axial images were reconstructed with the following settings: slice thickness, 1.0 mm; slice interval, 3.0 mm; and a bone kernel (FC30) with an AIRD (adaptive iterative dose reduction) 3D algorithm (Canon Medical Systems). The CT scanner was calibrated daily using a phantom.

### Measurement of HU values

**Fig. 1:**
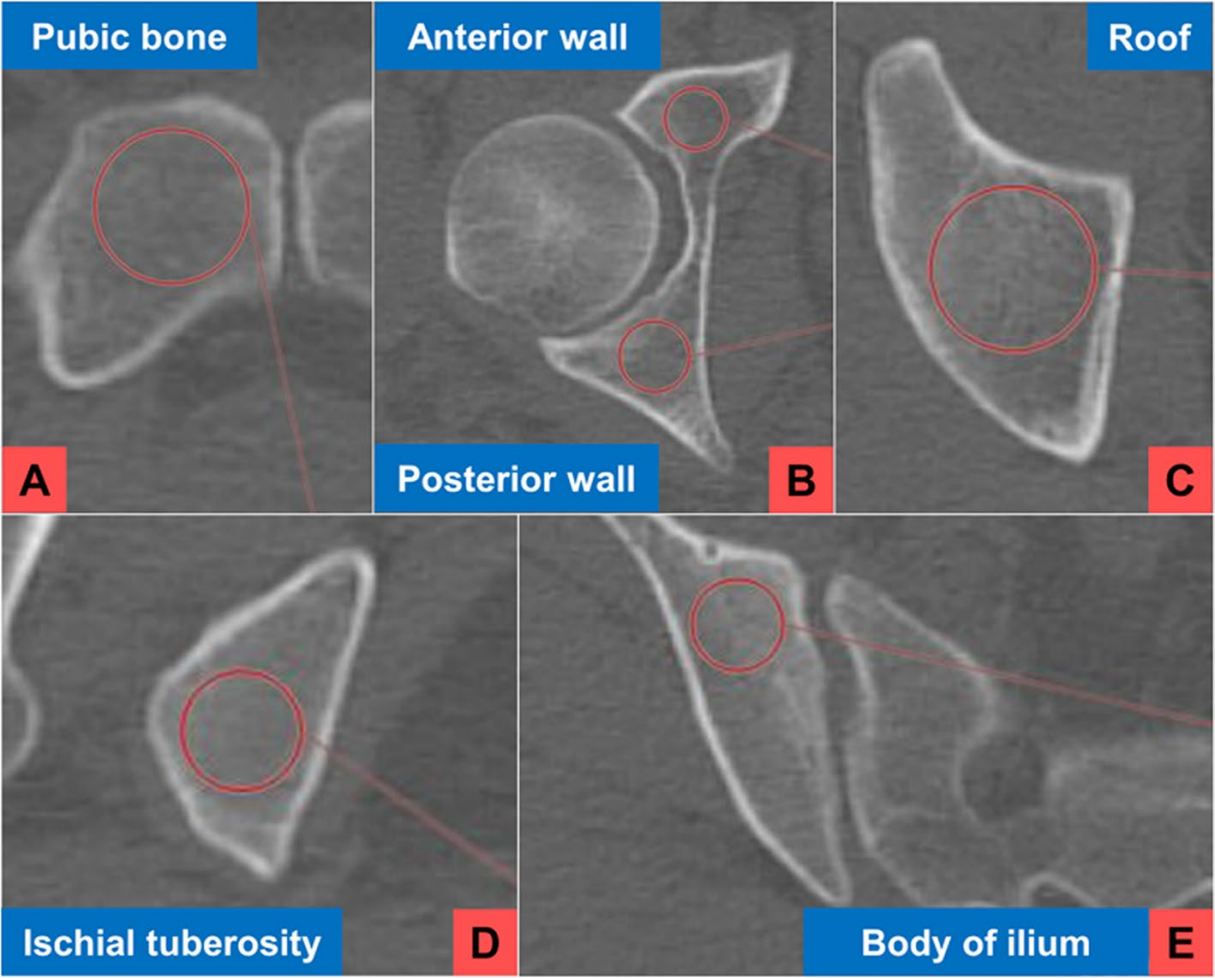
The regions of interest created in 6 areas of the pelvis. (**A**) Lateral to the pubic symphysis, (**B**) at the level of the center of the femoral head, (**C**) 4 mm above the articular surface, (**D**) at the ischial tuberosity, and (**E**) at the level of the second sacral foramina

The HU values were measured using ziostation2 software version 2.9.2.2 (Ziosoft Inc., Belmont, CA, USA). The HU values in the corresponding pelvic areas on the contralateral side to the fracture and the value at the third lumbar vertebra (L3) were measured in both groups. The axial CT image data were divided into 6 areas of the pelvis and L3. A region of interest (ROI) was created as a perfect circle 1 mm inside the cortical bone in each area. Measurements were obtained from cancellous regions only, with avoidance of cortical regions and degenerative changes [14, 22]. The ROI in the pubic bone was defined at the area lateral to the pubic symphysis, the ROIs of the anterior and posterior wall of the acetabulum were defined at the level of the center of the femoral head, the ROI of the acetabular roof was defined at 4 mm above the articular surface, that of the ischial tuberosity was defined at the ischial tuberosity, that of the body of the ilium was defined at the level of the second sacral foramina, and that of L3 was defined at the middle of the vertebral body (Fig. 1). All measurements were obtained in triplicate every other week and averaged by two investigators. These investigators who defined the bony landmarks were an experienced pelvic surgeons and trauma surgery resident. The reliability of this method was assessed using the intraclass correlation coefficient (ICC). The HU value measured in each area was compared between the young and older groups and according to sex.

### Statistical analysis

Quantitative data were expressed as the mean and the standard deviation. The primary outcome was the CT-based bone density of each areas of pelvic bone measured in HU. A one-way repeated measure ANOVA with a post-hoc Tukey–Kramer test was used to compare the HU values of each pelvic area with intragroup. The Mann–Whitney *U* test was used to assess differences in HU measurements in each area within and between the groups and according to sex. The intra-rater and inter-rater reliability of continuous data was determined by the ICC. All data were analyzed using JMP software version 14.2 (SAS Institute Inc., Cary, NC, USA). A p-value ≤ 0.05 was considered statistically significant.

## Results

Sixty-one young patients (32 men, 29 women) and 154 older patients (66 men, 88 women) were included in the study. Mean age was 31.9 ± 7.9 (range, 20–44) years in men in the young group, 31.9 ± 6.5 (range, 20–43) years in women in the young group, 77.7 ± 6.3 (range, 66–88) years in men in the older group, and 77.9 ± 6.1 (range, 65–89) years in women in the older group. There was no significant difference in age between men and women in either study group. The mean HU values for the six pelvic regions and L3 were shown in Table 1. The ICC of the intra-observer measurements for all each pelvic area by two investigators were 0.85–0.96 and 0.89–0.98, respectively. The ICC of the inter-observer measurements were 0.81–0.97.

**Table 1:** HU values recorded in each pelvic area in the study groups (mean ± standard deviation)

	Pubis	AW	Roof	PW	IT	BI	L3
Men in the young group	131 ± 37	134 ± 37	235 ± 44	179 ± 46	217 ± 48	219 ± 46	192 ± 34
Men in the older group	35 ± 29	37 ± 31	120 ± 34	89 ± 35	109 ± 36	109 ± 32	86 ± 28
Women in the young group	121 ± 33	118 ± 39	230 ± 34	172 ± 29	210 ± 39	210 ± 35	190 ± 28
Women in the older group	32 ± 29	26 ± 28	118 ± 50	76 ± 36	95 ± 31	92 ± 31	76 ± 24

*AW* Anterior wall, *BI* Body of the ilium, *IT* Ischial tuberosity, *L3* Third lumbar vertebra, *Pubis* Pubic bone, *PW* Posterior wall

**Fig. 2:**
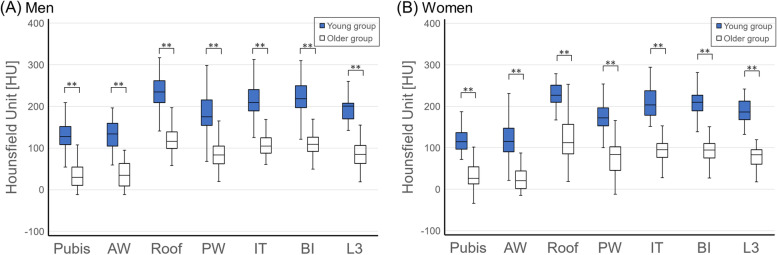
Comparison of Hounsfield unit measurements in the pelvis between (**A**) men and (**B**) women in the young group and the older group. **p < 0.01, Mann–Whitney *U* test. AW, anterior wall; BI, body of the ilium; IT, ischial tuberosity; L3, third lumbar vertebra; Pubis, pubic bone; PW, posterior wall

The HU value of each pelvic area was significantly different. The highest HU value was recorded for the roof of the acetabulum. HU values were significantly higher in the ischial tuberosity and body of the ilium and lower in the anterior part of the pelvis (pubic bone and anterior wall) than in the posterior part of the pelvis (posterior wall and body of the ilium) independent of age and sex by performing a one-way repeated measure ANOVA with a post-hoc Tukey–Kramer test. The ratios of HU values in the older group to those in the young group in the pubic bone, the anterior wall, roof, and posterior wall of the acetabulum, the ischial tuberosity, the body of the ilium, and L3 were (men 26%, women 27%), (28%, 22%), (51%, 51%), (50%, 44%), (51%, 45%), (50%, 44%), and (45%, 40%), respectively. The HU values in all pelvic areas were lower in the older group than in the young group independent of sex (Figs. 2 and 4).

**Fig. 3:**
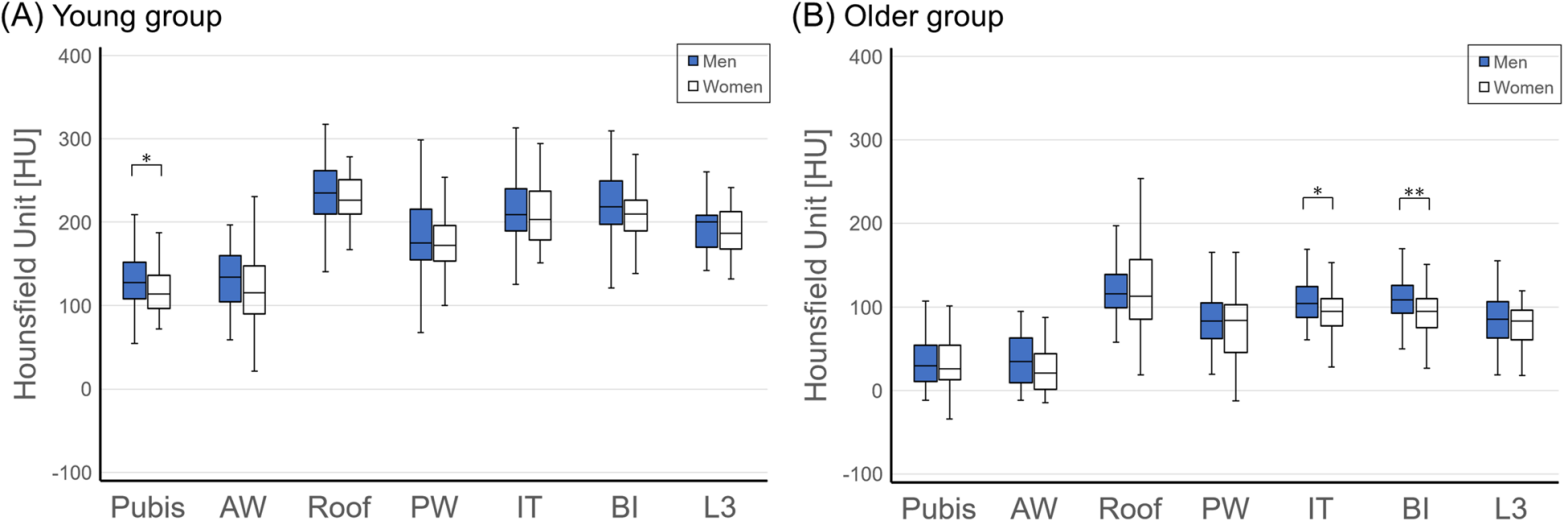
Comparison of Hounsfield unit measurements in the pelvis between men and women (**A**) in the young group and (**B**) in the older group. *p < 0.05, **p < 0.01, Mann–Whitney *U* test. AW, anterior wall; BI, body of the ilium; IT, ischial tuberosity; L3, third lumbar vertebra; Pubis, pubic bone; PW, posterior wall

Although all HU values were lower in the female pelvis than in the male pelvis, there was a statistically significant sex difference in the values recorded for the pubic bone in the young group and the ischial tuberosity and body of the ilium in the older group (Fig. 3).

## Discussion

### Distribution of HU values

To our knowledge, this study is the first to investigate the distribution of HU values in the pelvic bones. Our findings show that the HU values are not uniformly distributed throughout the pelvis. The HU values ranged from > 200 to < 120 in the young group. The highest value was found in the roof of the acetabulum. This finding was consistent, independent of age and sex, and predictable considering the effect of load on development and maintenance of bone mass and strength [23–25]. A previous biomechanical study showed that strain from loading was concentrated in the supra-acetabular region [26]. Moreover, area of the roof mostly affected by the weight-bearing load was within 45 degrees of the roof arc angle [27]. In our study, we found higher HU values at the ischial tuberosity and the body of the ilium. It has been demonstrated that the ischial tuberosity is subjected to physical stress during sitting and lying in the supine position [28]. Load is transmitted not only from bone to bone but also via the ligaments. The sacrotuberous ligament attached to the ischial tuberosity is strong and supports the load through the lower limb to the trunk muscles [29]. This ligament is subjected to approximately 28% of the load borne by the lower limb [30]. Higher HU values were also recorded for the body of the ilium. The sacroiliac joint, which includes the sacrum and ilium, bears the weight of the spine, and is responsible for transmitting weight into the lower extremities [31]. Ligaments at the back and front of this joint provide stability and share the stress. Hammer et al. reported that the anterior sacroiliac and iliolumbar ligaments were subjected to approximately 13–15% of the load [30]. Physical stress increases bone formation, resulting in increased BMD [32], which may account for the higher HU values at the ischial tuberosity and body of the ilium. In contrast, the pubic ligament was subjected to almost no loading in both the standing and sitting positions, resulting in a lower HU value in the pubic bone. Kotzar et al. found that the force along the lower limbs was always directed posteriorly to the acetabulum during all phases of walking [33]. The HU value was lower in the anterior wall than in the posterior wall in this study.

### Effect of age

**Fig. 4:**
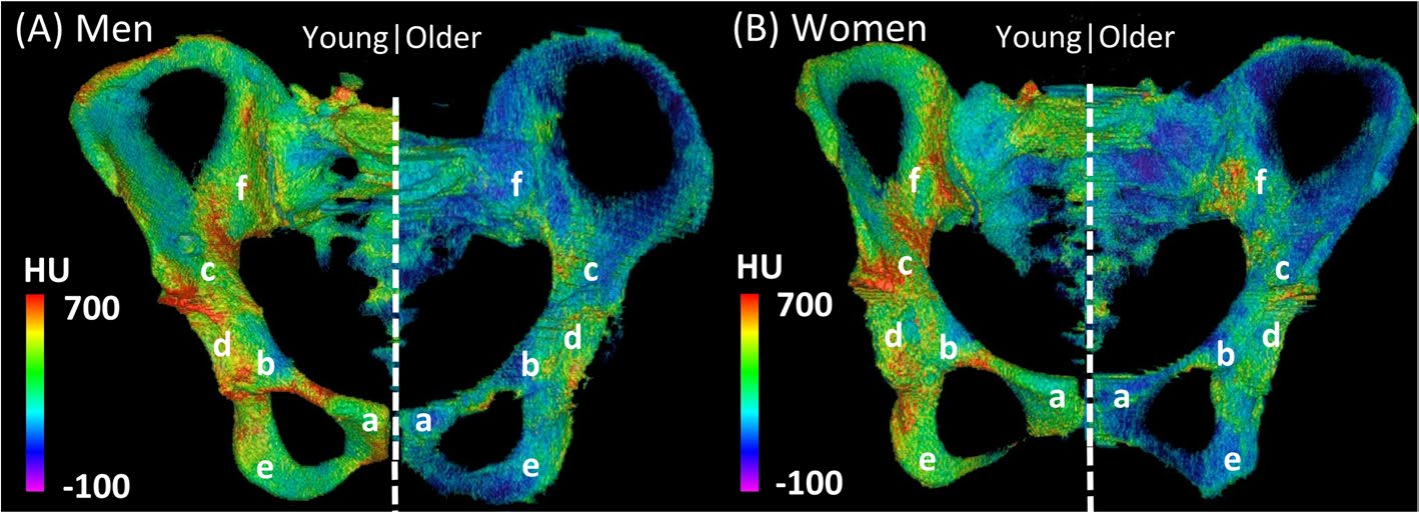
Three-dimensional models showing the distribution of Hounsfield unit (HU) values in the cancellous bone of the pelvis. The models were created using Osirix imaging software. (**A**) shows representative male cases (a 30-year-old man and a 71-year-old man) and (**B**) shows representative female cases (a 25-year-old woman and a 76-year-old woman). The color scale of Hounsfield unit values is displayed in the range of -100 to 700. a, pubic bone; b, anterior wall; c, roof; d, posterior wall; e, ischial tuberosity; f, body of the ilium

Aging greatly influenced the HU values recorded in this study. The HU values for all pelvic areas were significantly lower in the older group than in the young group (Figs. 2 and 4). The HU values in the anterior pelvic area were particularly low in the older group. Lee reported an HU cut-off value of 105 for osteoporosis at L3 [16]. Applying this cut-off value in our study, 97% of the women and 76% of the men in the older group had osteoporosis. Schönenberg et al. reported a mean HU value of 85 in elderly patients with a fracture in the body of S1 [19]. In contrast, the HU values in the anterior part of the pelvis in the older group were extremely low (pubic bone, 35 in men and 32 in women; anterior wall, 37 in men and 26 in women), suggesting a high risk of anterior pelvic fractures. This finding could explain the earlier reports of an acetabular fracture involving displacement of the anterior fragment being the most frequent pelvic fracture pattern in the elderly [2, 6]. However, the HU values in the young group were not low (pubic bone, 131 in men and 121 in women; anterior wall, 134 in men and 118 in women). Therefore, fractures involving the anterior fragment are likely to be unfrequented in young patients.

### Effect of sex

BMD is lower in women than in men and women lose relatively more bone mass with age [34, 35]. In this study, the HU values in all 6 pelvic areas were lower in women than in men regardless of age. However, a statistically significant difference was found in only 1 area in the young group and 2 areas in the older group (Fig. 3). This finding indicates that the effect of sex on pelvic BMD is less than that of age.

### Surgical treatment

Our results may help to guide the treatment of pelvic fractures because the density of cortical and cancellous bone has been directly associated with screw purchase and pullout strength [9, 10]. Bredow et al. reported that use of pedicle screws for vertebrae with HU values ≤ 120 resulted in screw loosening [36]. In our study, the HU values were ≤ 120 in all pelvic areas in the older with the exception of the roof. Fixation of pelvic fractures using implants that rely on screw anchorage is a clinical problem in the older. Screw fixation through the contralateral cortical bone is stronger than that in cancellous bone [37]. Therefore, in theory, screw anchorage through cortical bone on the contralateral side to the fracture could be considered and has been used by spine surgeons to guide the cortical bone trajectory and increase the pullout strength and stability of the screw [38].

### Limitations

There are some limitations to consider when interpreting the results of this study. First, Engelke et al. found that HU values differ by 10%-20% between different scanners [39]. However, we found that evaluation using the same CT scanner was reliable. Second, we measured HU values of L3 for comparison. L3 was selected, as this was the highest vertebral body that was included in the pelvic CT, with less degenerative changes compared to the lower lumbar vertebrae. Third, we measured HU values in only 6 selected pelvic areas. Technically, it would have been too difficult to measure the values in the entire pelvic region. In terms of load transmission to the lower extremities, the important areas in the pelvis are the roof of the acetabulum and its anterior and posterior walls. Moreover, the most anterior, posterior, and inferior parts of the pelvis (i.e., the pubic symphysis, ilium adjacent to the sacroiliac joint, and ischial tuberosity, respectively) are areas often used for screw insertion and are attachment sites for major ligaments. Fourth, different causes contribute to the development of fragility fractures. The greatest challenge in studying fragility fractures is targeting fragile patients before the development of a fracture, and current diagnostic tools suffer from limitations. Data from older patients without fragility fractures would be ideal, but CT examination is not possible in these patients due to radiation exposure. Thus, it is necessary to keep in mind that the older patients in this study were not representative of their age cohort. This study compared older adults with low-energy fragility fractures and young adults with high-energy fractures. Therefore, the effect of age may be exaggerated in this case. Fifth, the HU values recorded in each pelvic area may be affected by changes in spinopelvic alignment with age. For example, pelvic tilt may alter the loading distribution. This will be a subject for future study.

## Conclusions

In this study, we measured the regional HU values around the acetabulum and pelvic ring and compared them between young and older patients. We found that the highest HU value was at the roof and the lowest at the pubic bone and anterior wall. This finding was consistent regardless of age and sex. The HU distribution in the pelvis was strongly correlated with the loading applied to each area. Aging had a substantial effect on the HU values, especially in the anterior areas. The effect of sex was smaller than that of aging. These results may contribute to understanding acetabular fracture patterns and surgical treatment.

## Data Availability

The datasets used and analyzed during the current study are available from the corresponding author on reasonable request.

## Abbreviations

BMD: Bone mineral density
HU: Hounsfield unit
CT: Computed tomography
AEC: Automatic exposure control
SD: Standard deviation
L3: Third lumbar vertebra
ROI: Region of interest
ICC: Intraclass correlation coefficient
AW: Anterior wall
BI: Body of the ilium
IT: Ischial tuberosity
Pubis: Pubic bone
PW: Posterior wall
